# Optimization of the Extraction Conditions for *Buddleja officinalis* Maxim. Using Response Surface Methodology and Exploration of the Optimum Harvest Time

**DOI:** 10.3390/molecules22111877

**Published:** 2017-11-01

**Authors:** Guoyong Xie, Ran Li, Yu Han, Yan Zhu, Gang Wu, Minjian Qin

**Affiliations:** Department of Resources Science of Traditional Chinese Medicines, State Key Laboratory of Natural Medicines, China Pharmaceutical University, Nanjing 210009, China; guoyongxie321@163.com (G.X.); 18019553302@163.com (R.L.); 15951088918@163.com (Y.H.); cpuzy@126.com (Y.Z.); woosmail@163.com (G.W.)

**Keywords:** *Buddleja officinalis* Maxim, multiple responses optimization, optimum harvest time, response surface methodology

## Abstract

The Box-Behnken design was used to evaluate the effects of the methanol concentration (60–100%), liquid to solid ratio (20:1 to 40:1 mL/g) and extraction time (20–40 min) on the yield of 11 constituents from *Buddleja officinalis* Maxim using ultrasound-assisted extraction. The Derringer’s desirability function approach showed that the modified optimum extraction conditions were: 76% methanol concentration, 33 min extraction time and a 34:1 mL/g solvent to solid ratio. Under these conditions, the experimentally measured yields of the compounds were in good agreement with the predicted values. An accurate and sensitive method was also established using high-performance liquid chromatography with diode-array detection for the simultaneous determination of the 11 compounds in *Buddleja officinalis*. The newly developed method was used to determine the amounts of bioactive components in *Buddleja officinalis* during four different growth stages. According to these results, we recommend that the full blossom stage is the best time for harvesting this plant to obtain the highest yield of crude materials.

## 1. Introduction

*Buddleja officinalis* Maxim, a shrub in the family Loganiaceae, is distributed widely in the southwest and central regions of China. It was first recorded in “*Kai Bao Ben Cao*”, a one thousand year-old work on traditional Chinese medicines. *Buddleja officinalis* is known as “*Mi Meng Hua*” in Chinese and it is listed in the Chinese Pharmacopoeia. The flower buds and inflorescences of this medicinal plant are commonly used for the treatment of eye diseases, including conjunctival congestion and soreness, photophobia, delacrimation, and cloudy and blurred vision, as well as hepatic asthenia [1]. Recent research has shown that the main components of *B. officinalis* are flavonoids, phenethyl alcohol glycosides, and saponins [2,3,4,5], although alkaloids and carotenoids are also present in the plant. Linarin is the major flavone and an important bioactive constituent of this plant, and it was used as the index component of *B. officinalis* in the 2015 edition of the Chinese Pharmacopeia. A small number of other constituents (acteoside, luteolin, etc.) have been also considered for quality control for *B. officinalis* in earlier reports [6,7], but those studies had some weaknesses. As is well known, Chinese herbal medicines typically have a variety of chemical components and the ingredients may have synergistic or antagonistic effects. One or two chemical components, therefore, do not reflect the true efficacy of the herbal medicine and establishing a quality evaluation system for the multi-index constituents of *B. officinalis* is thus very necessary. To date, a method for the simultaneous quantification of multiple major compounds of *B. officinalis*, including flavonoids, phenethyl alcohol glycosides, alkaloids, and carotenoids has not been reported.

It is widely acknowledged that optimal extraction methods are very important in the establishment of a multi-index component determination system. Both conventional (cold soak, reflux and Soxhlet) extraction methods and non-conventional (microwave-assisted and ultrasound-assisted) extraction methods have been used to extract active constituents from natural products. The optimal extraction method is selected using factors such as the extraction efficiency and the time taken for the extraction process. Many extraction parameters (e.g., number of extractions, solvent composition, extraction time and liquid to solid ratio) significantly affect the extraction efficiency of crude medicines.

The response surface methodology (RSM) is a statistical method for optimizing multivariate data, which is used widely to optimize extraction process [8]. The principle of RSM is to establish a multiple quadratic regression equation to fit the functional relationships between factors and response values, and to then find the optimal process parameters by analyzing the regression equation [9]. The function of RSM and single factor tests is similar, but fewer experiments are needed for RSM, thereby reducing the usage of solvents and time is required, RSM can also provide sufficient information on interactions between factors, whereas single factor tests cannot [10]. Optimization of the extraction conditions for *B. officinalis* has been reported previously [11,12] but these studies only described the optimum conditions for the extraction of the total flavonoids or yellow pigments, and they did not identify single components. In the present study, the extraction of 11 active ingredients of *B. officinalis* was simultaneously optimized for the first time.

The quality of Chinese herbal medicine is also influenced by different plant development stages and harvest time. In general, the harvest time has a great influence on the quality of medicinal materials. As illustrated by the old Chinese saying, “In March, Yinchen is a herb but useless straw in April, and firewood in May” [13], the active components in Yinchen change during different seasons. Therefore, in the present study we attempted to identify the best harvest time for *B. officinalis*.

The objectives of the present study were to optimize the extraction process, establish an HPLC-DAD method for quantitative analysis, and determine the best harvest time for *B. officinalis*.

## 2. Results and Discussion

### 2.1. Preliminary Experiments

**Figure 1:**
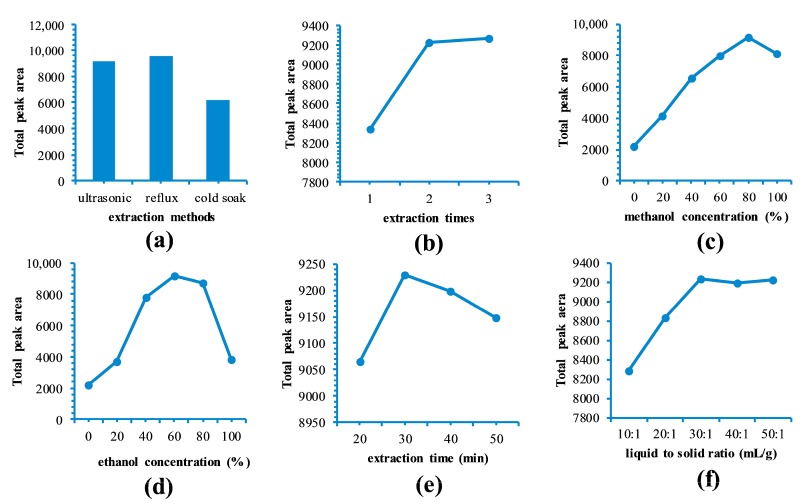
Results of single factor experiments (total peak area for 11 compounds): (**a**) extraction methods; (**b**) extraction times; (**c**) methanol concentration (%); (**d**) ethanol concentration (%); (**e**) extraction time (min); (**f**) liquid to solid ratio (mL/g).

The effect of the extraction method on the extraction yield (total peak area for 11 compounds) from *B. officinalis* was determined. Three extraction methods were evaluated: refluxing (80 °C) twice for 1 h with 80% (*v*/*v*) aqueous methanol (20 mL), ultrasound-assisted extraction (UAE, 100 W) for 30 min at ambient temperature, and cold soak for 12 h at ambient temperature. The extraction yields were in the following order: ultrasound-assisted ≈ reflux > cold soak (Figure 1a). Considering the convenience of the experimental protocols, UAE was selected as the preferred method.

The effect of extraction times on extraction efficiency was studied under the following conditions: 80% methanol concentration, liquid to solid ratio of 40:1 mL/g and UAE for 30 min. The extraction efficiencies for the first, second, and third extractions were 90%, 9%, and 1%, respectively (Figure 1b), thereby demonstrating that two extractions were sufficient to achieve the maximum extraction efficiency.

The impact of the extraction solvent and solvent proportion on the extraction efficiency was investigated by extracting twice for 30 min using UAE, with a liquid to solid ratio of 40:1 mL/g. The amount of material extracted was maximized at around 60–80% alcohol but it then decreased (Figure 1c,d). The largest total peak area was achieved using 80% (*v*/*v*) aqueous methanol so this solvent was selected for further experiments.

Samples were then extracted twice using UAE for different extraction time, with a methanol concentration of 80% and a liquid to solid ratio of 40:1 mL/g. The total peak area increased gradually as the extraction time increased from 20 to 30 min, but then decreased slightly when the extraction time exceeded 30 min (Figure 1e). The observed decrease in yield may have been due to substance degradation caused by the longer extraction times [14]. Thus, an extraction time of 30 min was considered suitable for extraction.

A series of trial extractions of *B. officinalis* was conducted by extracting twice for 30 min using UAE, with a methanol concentration of 80% and different liquid to solid ratios. The extraction yield reached a maximum value at 30:1 mL/g and then remained constant at this level (Figure 1f). Typically, a high ratio of liquid to solid improves the extraction yield because substances are dissolved more effectively [15]. Based on these preliminary experiments, a methanol concentration of 60–100%, a liquid to solid ratio between 20:1 and 40:1 mL/g, and an extraction time of 20–40 min were used in the RSM experiment to determine the optimum conditions for the extraction process, in which the extraction rates for the 11 compounds were used as responses.

### 2.2. RSM Optimization

#### 2.2.1. Fitting the Model

The 17 designed experiments and the corresponding responses are shown in Table 1. Based on the analysis, 11 regression equations were obtained (Table 2) to describe the empirical relationships between the independent and dependent variables. ANOVA was used to verify the 11 models (Table S1).

As an example, for Y_7_ (linarin), the significance and suitability of the model (model *p*-value, lack of fit, determination coefficient (*R*^2^), adjusted coefficient of determination (adj. *R*^2^), adequate precision, and CV) were determined by ANOVA and a statistical summary is presented in Table 3. The high *F*-value (22.74) and low *p*-value (0.0002) suggest that the calculated model was highly significant and suitable for this experiment. The lack of fit was non-significant, with a *p*-value of 0.0527 (i.e., >0.05). Moreover, the value of *R*^2^ was relatively high (0.9669), thereby indicating that only 3.31% of the changes in the response values could not be accounted for by the model [14,16]. However, these parameters are not sufficient to show that the model is reasonable and reliable for predicting the extraction yield and additional statistical parameters are necessary.

The proposed model had a high adj. *R*^2^ value (0.9244), which suggests that insignificant terms were excluded from the model [17,18]. The CV value was 3.12 (CV < 10 gives better reproducibility) and the precision value was 14.108 (>4 indicates adequate model discrimination). All of these statistical parameters demonstrate that the response could be estimated effectively using the model. In general, the model term is more significant when the *p*-value is smaller. For this example (Y_7_), X_1_, X_2_, X_1_X_3_, and X_1_^2^ were significant model terms, with *p*-values < 0.05. The quadratic term for the methanol concentration (*p* < 0.0001, negative effect) had the largest effect, followed by the linear terms X_1_ (*p* = 0.0001, negative effect) and X_2_ (*p* = 0.0016, positive effect), and then the interaction term X_1_X_3_ (*p* = 0.0170, positive effect).

Three-dimensional (3-D) response surface plots generated by keeping one factor at the central value and adjusting the other two variables within their test ranges were used to depict the effects of the three factors on the dependent variables. A red color on the 3-D plot indicates that the response is increasing [19] and an elliptical contour plot indicates that the interaction between the variables is significant [20].

**Figure 2:**
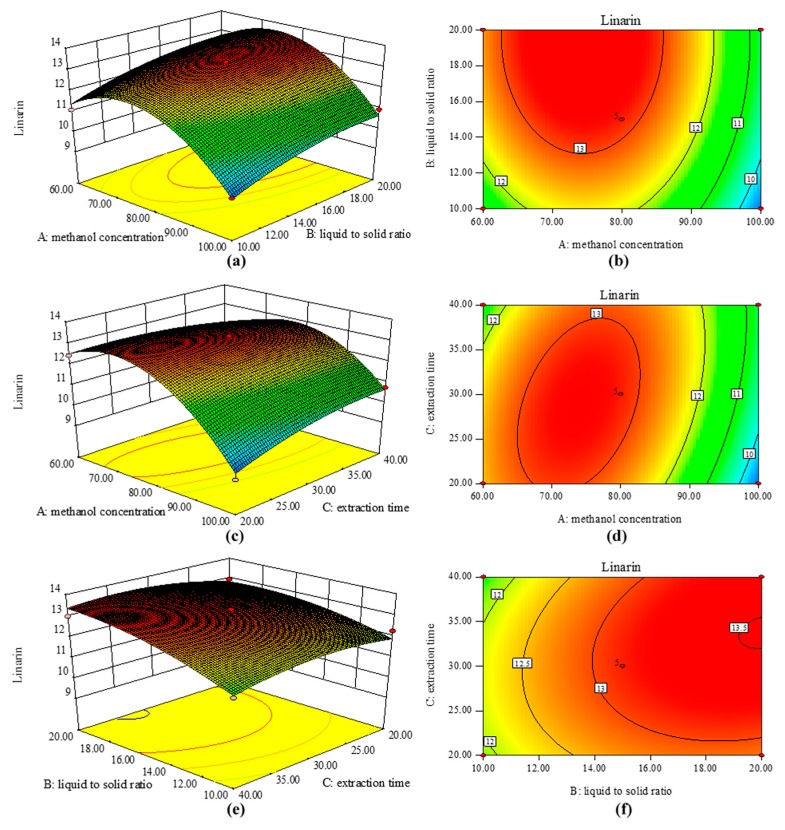
Response surface graphs of linarin: methanol concentration versus liquid/solid ratio (**a**,**b**); methanol concentration versus extraction time (**c**,**d**) and liquid/solid ratio versus extraction time (**e**,**f**).

The response surface plots for linarin (Y_7_) are shown in Figure 2. The plot/contours at varying concentrations and liquid to solid ratios with the time fixed at 30 min (0 level) are shown in Figure 2a,b. 

Initially, the yield of linarin increased with the methanol concentration, but it then declined sharply at higher concentrations, where the shape of the contour resembled a downward parabola. As the liquid to solid ratio increased, there was an increase in the yield of linarin, which then remained at a steady level. Higher amounts of linarin were obtained with a methanol concentration in the range 65–80% and a liquid to solid ratio >28:1 mL/g. The relationships between the methanol concentration and extraction time are shown in Figure 2c,d. The methanol concentration had a strong influence on the yield of linarin. A similar result (downward parabola) was observed for the extraction time but the effect was less marked than that for the methanol concentration. In general, we can conclude that the effect of the interaction term X_1_X_3_ was significant and that of the extraction time X_3_ was not, as shown by the corresponding results in Table 3. The interactions between the effect of the liquid to solid ratio and the extraction time on the yield of linarin at a fixed methanol concentration of 80% (0 level) are shown in Figure 2e,f, which indicate that the maximum yield was obtained with a liquid to solid ratio >28:1 mL/g and an extraction time of 33 min. The influence of the extraction time was not great compared with the effects of the methanol concentration and the liquid to solid ratio. Other studies have also shown that the time factor is not significant in UAE [19,21], possibly because ultrasonication causes the rapid dissolution of the chemicals.

#### 2.2.2. Optimization of Multiple Responses

**Figure 3:**
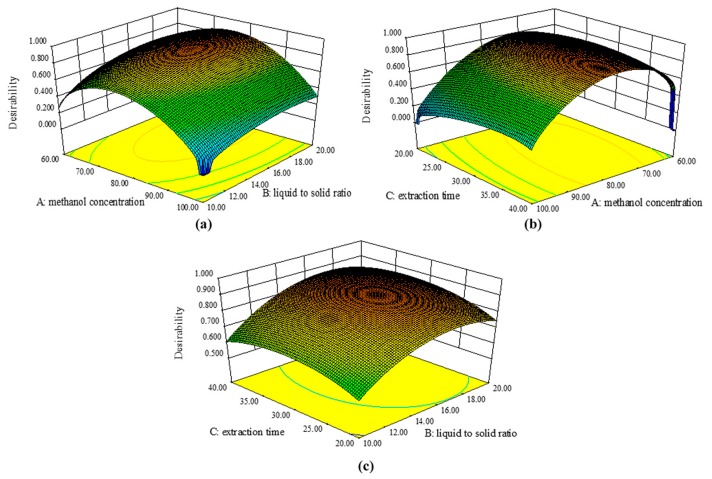
Response surface plots corresponding to the desirability function (maintaining one of the variables at optimum values): (**a**) methanol concentration-liquid/solid ratio; (**b**) extraction time-methanol concentration; (**c**) extraction time-liquid/solid ratio.

In general, optimizing the conditions for a single response is easy but we considered 11 responses in our study. The most widely used method for the simultaneous optimization of multiple responses is Derringer’s desirability function approach, which is based on the premise that the quality of a process with multiple quality characteristics is completely unacceptable when one of the characteristics is outside of the desired limits. This approach can determine the most desirable response values by setting an optimum condition, where the procedure involves constructing an overall desirability function D, which is a weighted geometric mean of the individual desirabilities (d_i_) [22]. When the value of the overall desirability function D is close to 1, this represents a completely desirable or ideal response value, whereas values of D close to 0 denote the opposite. The optimal conditions were calculated by numerical optimization, which sets goals for each response (11 responses set at the “maximum”) and independent variables (three variables set at “in range”). One solution was found where the ideal extraction conditions comprised a methanol concentration of 76%, a liquid to solid ratio of 34.1:1 mL/g, and an extraction time of 32.79 min. Based on the optimum process conditions, the predicted values for the 11 compounds are tabulated in Table 4. The 3-D plots obtained for the overall desirability function D by maintaining one parameter at the predicted value are shown in Figure 3. When the region in the vicinity of the optimal condition is quite flat, this means that small changes in the predicted value will not drastically affect the overall desirability.

#### 2.2.3. Authentication of Predicted Model

To verify the fitness of the model equation, a verification experiment was conducted under adjusted extraction conditions (methanol concentration, 76%; liquid to solid ratio, 34:1 mL/g; and extraction time, 33 min). The experimental values are shown in Table 4. The experimental values were close to the predicted values for all 11 compounds, where the relative errors were in a reasonable range. Thus, the RSM model was used to predict the optimal response values in the validation experiments.

### 2.3. Validation of the HPLC-DAD Method

**Figure 4:**
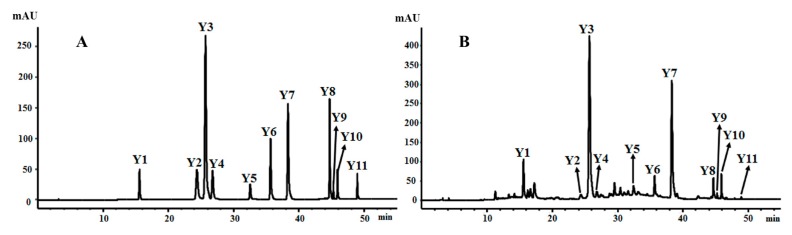
HPLCs of authentic standards and methanol extract of *Buddleja officinalis* flowers. (**A**) UV chromatogram of the 11 authentic standards at 330 nm; (**B**) UV chromatogram of methanol extract at 330 nm. (1) echinacoside (Y_1_); (2) luteolin-7-*O*-rutinoside (Y_2_); (3) acteoside (Y_3_); (4) luteolin-7-*O*-glucoside (Y_4_); (5) apigenin-7-*O*-glucuronide (Y_5_); (6) neobudofficide (Y_6_); (7) linarin (Y_7_); (8) *N*^1^, *N*^5^, *N*^10^-(*E*)-tri-*p*-coumaroylspermidine (Y_8_); (9) crocin III (Y_9_); (10) apigenin (Y_10_); (11) acacetin (Y_11_).

The newly developed chromatography method was validated based on the linearity, limit of detection (LOD), limit of quantification (LOQ), precision, repeatability, stability, and accuracy. HPLC chromatograms of the mixed standard stock solution and an extract of *B. officinalis* are shown in Figure 4.

#### 2.3.1. Linearity, LOD and LOQ

Eleven calibration curves were produced by plotting the different concentrations (*X* axis, mg/mL) against the corresponding mean peak areas (*Y* axis), and the regression equations were calculated using the least squares method. The coefficients of determination (*R*^2^) were all >0.9990 (Table 5), which indicated that the method exhibited good linearity for all of the analytes within the tested range. The LOD values for the 11 reference compounds were in the range of 24.7–63.2 ng/mL and the LOQ values were in range of 61.3–218.1 ng/mL, thereby indicating that the method was sensitive for all 11 compounds.

#### 2.3.2. Precision

The relative standard deviation (RSD) values for intra-day precision were <1.63% and those for inter-day precision were <2.94% (The RSD of the proposed requirement is less than 5%), which indicates that the precision of the instrument is good.

#### 2.3.3. Repeatability

The RSD values for all the compounds were in range of 0.91–4.27%, this means that the method has good reproducibility.

#### 2.3.4. Stability

The sample had good stability in the 48 h stability experiment with RSD values of 11 constituents <3.32%.

#### 2.3.5. Accuracy

The results of the recovery test showed that the mean recovery rates were between 95.80% and 102.81%, with RSD values in the range of 0.98–2.50%, which indicates that the method is accurate. The results for all parameters demonstrated that the proposed method was both accurate and reliable for analyzing *B. officinalis.* Compared with the previously proposed analytical methods, our method has the advantage that it can identify and quantify 11 different compounds simultaneously.

### 2.4. Analysis of Samples in Different Flowering Stages

The time of harvest has a large impact on the bioactive component contents of many traditional Chinese medicines, including *Lonicera japonica* Thunb and Herba *Artemisiae Scopariae* [13,23]. Thus, we investigated the dynamic changes in the 11 bioactive compounds during different flowering stages in order to determine the best time for harvesting *B. officinalis*.

**Figure 5:**
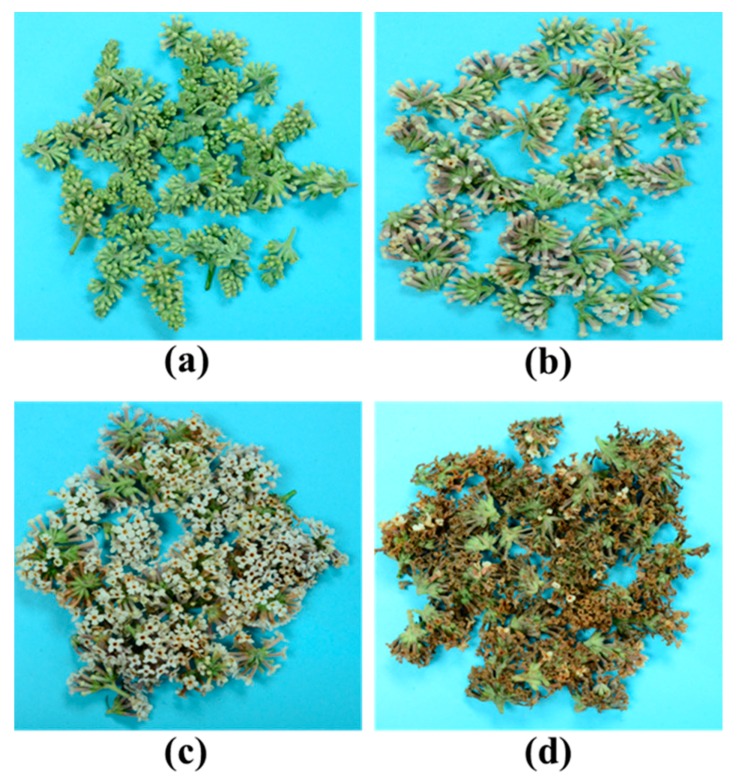
Fresh materials of *B. officinalis* at four different growth periods: (**a**) bud; (**b**) early flower; (**c**) full-blossom; (**d**) withering stage.

**Figure 6:**
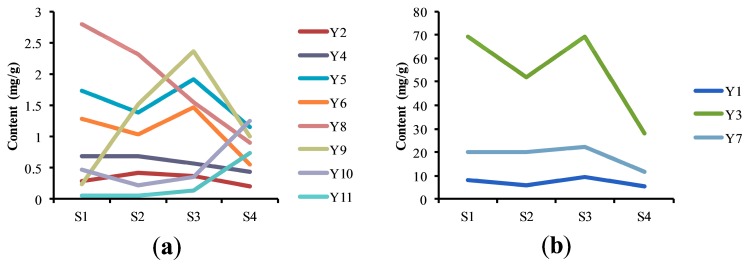
Changes in content of the eleven constituents in four different growth periods: (**a**) Y_2_/Y_4_/Y_5_/Y_6_/Y_8_/Y_9_/Y_10_/Y_11_; (**b**) Y_1_/Y_3_/Y_7_.

In terms of flower size, color, and morphology, the growth of *B. officinalis* can be divided into four different stages: bud stage (S1), early flower stage (S2), full blossom stage (S3), and withering stage (S4) (Figure 5). The composition of *B. officinalis* during different growth stages was analyzed using the established extraction method and chromatography conditions (Table S2). The changes in the amounts of Y_1_, Y_3_, Y_5_, Y_6_ and Y_7_ followed the shape of an inverted “N” (Figure 6), with maximum values at S3. The amount of linarin (Y_7_) in each stage was >0.5% (5 mg/g), which is the amount stipulated by the Chinese pharmacopoeia. The amounts of Y_4_ and Y_8_ decreased throughout growth, whereas the amounts of Y_10_ and Y_11_ reached a maximum at S4 (1.26 mg/g for Y_10_ and 0.73 mg/g for Y_11_). The highest amounts of Y_2_ and Y_9_ were observed at S2 and S3, respectively.

In summary, higher amounts of the most bioactive components were present in the full blossom stage. This applied particularly to crocin III (Y_9_), which has a variety of biological effects, including anti-inflammatory, antioxidant, antitumor, and hepatoprotective activities. It is also used to treat eye diseases since many of the bioactives are similar to those in *B. officinalis*. The level of Y_9_ (2.37 mg/g) during the full blossom stage was 10 times higher than that in the bud stage. The experimental data showed that another component in *B. officinalis*, acteoside (Y_3_), which also has significant bioactivity [24,25,26], was present in the greatest amounts. Thus, acteoside could have an important role in determining the quality of *B. officinalis*, although linarin is used as the only quality control index at present. The dynamic accumulation of acteoside during all four growth stages is remarkable and the contents are almost the same during S1 and S3. The most bioactive compounds are present at their highest levels during S3, so this stage may be the most appropriate for harvesting *B. officinalis* for medicinal use. Lanying et al., also reported that the flavonoid content is higher in the full blossom than the flower bud period [27], which again contradicts the Chinese Pharmacopoeia stipulation for using flower buds as the crude medicine. A possible explanation for this discrepancy is that the activities of the related enzymes are different during the four flowering periods [23]. Most enzymes will reach their maximum activity in full blossom and further studies are needed to explore the mechanistic reasons for this discrepancy.

## 3. Materials and Methods

### 3.1. Plant Samples

*Buddleja officinalis* Maxim samples (flower buds and inflorescences) used for optimizing the extraction conditions was purchased from Bozhou (Anhui Province, China). Samples during different flowering stages (bud, early flower, full blossom, and withering stage) were collected from Guizhou Province between March and April in 2016, and dried in the sun (according to the result of pre-experiments) until the moisture content was <12% (according to the stipulation for flower medicines in the Chinese Pharmacopoeia). Moisture content was determined using a Sartorius MA 35 rapid moisture meter (Sartorius AG, Göttingen, Germany) at 105 °C, using the method established in our laboratory. The experimental samples were ground into fine powders, sieved through a 60-mesh sieve, and then stored in a drier over silica gel.

### 3.2. Chemicals and Reagents

Reference substances, echinacoside (Y_1_), apigenin-7-*O*-glucuronide (Y_5_) and acacetin (Y_11_), were purchased from Baoji Herbest Bio-Tech Co. Ltd. (Baoji, China). Luteolin-7-*O*-rutinoside (Y_2_), acteoside (Y_3_), luteolin-7-*O*-glucoside (Y_4_), neobudofficide (Y_6_), linarin (Y_7_), *N*^1^,*N*^5^,*N*^10^-(*Z*)-tri-*p*-coumaroylspermidine (Y_8_), crocin III (Y_9_) and apigenin (Y_10_) were separated and purified (>98% by HPLC analysis) in our own laboratory and their structures were confirmed using MS and ^1^H-NMR and ^13^C-NMR spectroscopy. HPLC grade methanol and acetonitrile were purchased from Hanbon Sci. & Tech Co. Ltd. (Huaian, China) and Merck (Darmstadt, Germany), respectively. Purified water was obtained from Wahaha Group Co. Ltd. (Hangzhou, China). All of the other chemicals and solvents were analytical grade and were purchased from Nanjing Chemical Regents Co. Ltd. (Nanjing, China).

### 3.3. Ultrasound-Assisted Extraction (UAE)

Powdered samples (0.5 g) of *B. officinalis* were mixed with aqueous methanol (0–100%) using varying ratios of solvent to solid (10–50 mL/g) and extracted for different periods of time (20–50 min) at ambient temperature in 50-mL centrifuge tubes, before they were exposed to ultrasound using a KH5200DB ultrasonic cleaner (Kunshan Ultrasonic Instrument Co. Ltd., Kunshan, China). The mixtures were centrifuged at 1400× *g* for 10 min and the insoluble sludge was re-extracted. The supernatants were combined and diluted to 50 mL with the extraction solvent. Samples (1 mL) of the extracts were then filtered through a 0.22-µm microfiltration membrane before further analysis.

### 3.4. HPLC Analysis

HPLC analyses were conducted using an Agilent Series 1260 LC instrument (Agilent Technologies, Santa Clara, CA, USA) equipped with an autosampler, column temperature controller, DAD, quaternary pump, and online degasser. The analytes were separated using a Hanbon Megres C_18_ column (4.6 mm × 250 mm, 5 µm, Hanbon Sci. & Tech Co. Ltd., Huaian, China). The injection volume was 10 µL, the flow rate was 1.0 mL/min, and the column temperature was 25 °C. The detection wavelengths were set at 330 nm and 440 nm (maxima for crocin III) based on the the results obtained by full wave scanning. The mobile phase comprised solvent A (0.1% aqueous formic acid, *v*/*v*) and solvent B (acetonitrile) with the following elution gradient: 0–5 min (8–10% B), 5–10 min (10–18% B), 10–20 min (18–18.5% B), 20–34 min (18.5–30% B), 34–38 min (30–30% B), 38–41 min (30–37% B), 41–50 min (37–95% B), and 50–55 min (95–95% B). The total run time was 55 min and the time of column equilibration was 5 min. The mixed standard stock solution of 11 reference compounds was prepared by dissolving echinacoside (0.154 mg/mL), luteolin-7-*O*-rutinoside (0.061 mg/mL), acteoside (0.834 mg/mL), luteolin-7-*O*-glucoside (0.0255 mg/mL), apigenin-7-*O*-glucuronide (0.058 mg/mL), neobudofficide (0.1615 mg/mL), linarin (0.39 mg/mL), *N*^1^,*N*^5^,*N*^10^-(*E*)-tri-*p*-coumaroylspermidine (0.250 mg/mL), crocin III (0.254 mg/mL), apigenin (0.0625 mg/mL), and acacetin (0.029 mg/mL) in 60% aqueous acetonitrile. The solution was used for calibration and to calculate the linear correlation coefficient of the curve. The results were expressed as mg/g dry weight.

### 3.5. Validation of the HPLC-DAD Method

#### 3.5.1. Linearity, LOD and LOQ

The mixed standard stock solution containing the 11 reference compounds was prepared as described in Section 3.4. Then the standard solution was diluted to seven concentration levels using 60% acetonitrile to construct the calibration curves, and the linear relation is measured by the calculation of *R*^2^. The LOD and LOQ represent the lowest concentrations that can be detected at signal-to-noise ratios of 3 and 10, respectively.

#### 3.5.2. Precision

The intra-day precision was evaluated by injecting the same standard solution six times in one day and the inter-day precision was evaluated by injecting the same standard solution twice each day for three consecutive days.

#### 3.5.3. Repeatability

The repeatability was tested by analyzing five sample solutions in parallel, which were prepared using the method described in Section 3.3.

#### 3.5.4. Stability

The stability was determined by measuring the peak area of a fresh sample solution at different time points (0, 2, 4, 6, 8, 12, 24 and 48 h).

#### 3.5.5. Accuracy

The accuracy of the HPLC method was assessed by measuring the recovery rates. A previously analyzed sample of powder (0.25 g, accurately weighed) was spiked with the 11 standard compounds at 1 × concentration level (*n* = 5). Solutions were prepared as described in Section 3.3 and then analyzed.

### 3.6. Experimental Design

#### 3.6.1. Preliminary Experiments

In the preliminary experiments, five factors (extraction method, number of extractions, methanol/ethanol concentration, extraction time and liquid to solid ratio) were chosen for evaluation. Extraction methods were heat reflux, UAE and cold soak extraction; number of extractions was 1, 2 and 3; methanol/ethanol concentrations were 0%, 20%, 40%, 60%, 80% and 100%; extraction times were 20, 30, 40 and 50 min; liquid to solid ratios were 10:1, 20:1, 30:1, 40:1 and 50:1 mL/g.

#### 3.6.2. Response Surface Methodology

The conditions for the extraction of *B. officinalis* were optimized using RSM. The Box-Behnken design (BBD) was used to evaluate the effects of three independent variables (methanol concentration/X_1_, liquid-to-solid ratio/X_2_, and extraction time/X_3_) on the extraction yield of the 11 compounds from *B. officinalis*. Appropriate ranges for the methanol concentration (60–100%), liquid to solid ratio (20–40 mL/g), and extraction time (20–40 min) were selected based on the results of single factor tests. The three levels for each variable were coded as +1, 0 and −1 to denote high, middle, and low values, respectively. In total, 17 experimental runs were conducted, which comprised 12 factorial and five central point experiments. The results were fitted to a quadratic polynomial regression model as follows:

$$Y=b_{0}+\sum_{i=1}^{3}b_{i}X_{i}+\sum_{i=1}^{3}b_{ii}X_{i}^{2}+\sum_{i=1}^{2}\sum_{j=i+1}^{3}b_{ij}X_{i}X_{j}$$

where Y represents the predicted response and X_*i*_, X_*j*_ are the independent variables. The regression coefficients of the intercept, linear, quadratic, and interaction terms are defined as *b*_0_, *b_i_*, *b_ii_*, and *b_ij_*, respectively.

The data generated by the RSM experiments were analyzed using Design Expert version 8.06 (Stat-Ease Inc., Minneapolis, MN, USA). The adequacy of the model was assessed by analysis of variance (ANOVA). The BBD outputs also contained contour and three-dimensional surface response plots, which indicated the relationships between the responses and independent variables.

### 3.7. Validation of the Model

The validity of the model was demonstrated based on comparisons of the experimental values obtained under optimal conditions and the predicted values based on the coefficient of variation (CV, %).

### 3.8. Statistical Analysis

Assays were performed in triplicate and data are expressed as the mean value ± standard deviation. The data generated by the RSM experiments were analyzed using Design Expert version 8.06. The adequacy of the model was assessed by analysis of variance (ANOVA). The BBD outputs also contained contour and three-dimensional surface response plots, which indicated the relationships between the responses and independent variables.

## 4. Conclusions

RSM was used to successfully perform multi-response optimization of the extraction parameters for the 11 major components of *B. officinalis*. The model of the 11 responses was significant and the lack of fit was non-significant. For most of the responses, the effect of the methanol concentration was largest, followed by the solvent to solid ratio and extraction time. Derringer’s desirability function showed that the modified optimum extraction conditions were: methanol concentration, 76%; extraction time, 33 min; and solvent to solid ratio, 34:1 mL/g. A verification experiment was conducted using these conditions and there were no significant differences between the experimental and predicted values. We also established an accurate and sensitive HPLC method for the simultaneous determination of all 11 compounds in *B. officinalis*. The newly developed method was used to analyze *B. officinalis* during four different growth stages and we recommend that the full blossom stage is the best time for harvesting based on the results.

## Figures and Tables

**Table 1:** Experimental results of the Box-Behnken design (*n* = 3).

Run	Independent Variables ^a^			Dependent Variables ^b^										
	X_1_	X_2_	X_3_	Y_1_	Y_2_	Y_3_	Y_4_	Y_5_	Y_6_	Y_7_	Y_8_	Y_9_	Y_10_	Y_11_
1	80 (0)	15 (0)	30 (0)	5.02	0.99	27.25	0.60	0.98	2.30	13.30	1.45	0.98	0.60	0.13
2	80 (0)	10 (−1)	40 (1)	4.55	0.98	25.63	0.54	0.95	2.11	11.55	1.34	0.94	0.55	0.12
3	80 (0)	15 (0)	30 (0)	5.22	1.04	27.44	0.60	1.11	2.31	13.23	1.40	1.01	0.59	0.13
4	60 (−1)	15 (0)	20 (−1)	5.18	0.99	24.53	0.57	1.21	2.29	12.43	1.30	0.97	0.52	0.12
5	80 (0)	15 (0)	30 (0)	4.77	0.97	26.71	0.53	1.07	2.22	12.80	1.43	1.00	0.56	0.13
6	80 (0)	15 (0)	30 (0)	5.25	1.07	27.69	0.65	1.15	2.33	13.28	1.51	0.96	0.61	0.13
7	80 (0)	20 (1)	40 (1)	4.95	1.04	26.53	0.61	1.11	2.26	12.99	1.34	1.01	0.59	0.13
8	60 (−1)	20 (1)	30 (0)	4.45	1.09	23.31	0.74	1.35	2.26	12.67	1.25	1.01	0.53	0.13
9	60 (−1)	10 (−1)	30 (0)	4.20	1.07	21.00	0.74	1.27	2.06	11.08	1.22	0.91	0.43	0.11
10	100 (1)	10 (−1)	30 (0)	3.29	0.57	24.39	0.16	0.47	1.53	9.36	1.29	0.72	0.49	0.11
11	60 (−1)	15 (0)	40 (1)	4.62	1.09	22.72	0.75	1.39	2.23	11.92	1.21	0.99	0.49	0.13
12	80 (0)	15 (0)	30 (0)	5.06	1.03	27.07	0.57	1.15	2.29	13.19	1.49	1.01	0.59	0.14
13	100 (1)	20 (1)	30 (0)	4.03	0.64	25.89	0.18	0.61	1.74	10.94	1.36	0.74	0.50	0.12
14	100 (1)	15 (0)	20 (−1)	3.43	0.51	23.88	0.16	0.42	1.41	8.92	1.22	0.64	0.45	0.12
15	100 (1)	15 (0)	40 (1)	4.25	0.63	26.47	0.18	0.67	1.78	10.74	1.44	0.81	0.53	0.11
16	80 (0)	10 (−1)	20 (−1)	4.93	0.89	26.23	0.41	0.94	2.14	12.26	1.28	0.95	0.56	0.12
17	80 (0)	20 (1)	20 (−1)	4.80	0.94	26.57	0.51	1.05	2.22	12.92	1.29	1.00	0.58	0.13

^a^ Independent variables: X_1_, methanol concentration (%); X_2_, liquid/solid ratio (mL/0.5 g); X_3_, extraction time (min); ^b^ Dependent variables (mg/g): Y_1_, echinacoside; Y_2_, luteolin-7-*O*-rutinoside; Y_3_, acteoside; Y_4_, luteolin-7-*O*-glucoside; Y_5_, apigenin-7-*O*-glucuronide; Y_6_, neobudofficide; *Y*_7_, linarin; Y_8_, *N*^1^, *N*^5^, *N*^10^-(*E*)-tri-*p*-coumaroylspermidine; Y_9_, crocin III; Y_10_, apigenin; Y_11_, acacetin.

**Table 2:** Equations of 11 responses in terms of coded factors.

Compound	Equations ^a^
**1**	Y_1_ = 5.06 − 0.43X_1_ + 0.16X_2_ + 3.750 × 10^−3^X_3_ + 0.12X_1_X_2_ + 0.35X_1_X_3_ + 0.13X_2_X_3_ − 0.75X_1_^2^ − 0.32X_2_^2^ + 0.061X_3_^2^
**2**	Y_2_ = 1.02 − 0.24X_1_ + 0.025X_2_ + 0.051X_3_ + 0.013X_1_X_2_ + 5.000 × 10^−3^X_1_X_3_ + 2.500 × 10^−3^X_2_X_3_ − 0.17X_1_^2^ − 0.010X_2_^2^ − 0.047X_3_^2^
**3**	Y_3_ = 27.21 + 1.1X_1_ + 0.63X_2_ + 0.016X_3_ − 0.21X_1_X_2_ + 1.10X_1_X_3_ + 0.14X_2_X_3_ − 2.71X_1_^2^ − 0.87X_2_^2^ − 0.12X_3_^2^
**4**	Y_4_ = 0.59 − 0.26X_1_ + 0.024X_2_ + 0.054X_3_ + 5.000 × 10^−3^X_1_X_2_ − 0.040X_1_X_3_ − 7.500 × 10^−3^X_2_X_3_ − 0.12X_1_^2^ − 0.016X_2_^2^ − 0.056X_3_^2^
**5**	Y_5_ = 1.09 − 0.38X_1_ + 0.061X_2_ + 0.063X_3_ + 0.015X_1_X_2_ + 0.018X_1_X_3_ + 0.013X_2_X_3_ − 0.13X_1_^2^ − 0.039X_2_^2^ − 0.041X_3_^2^
**6**	Y_6_ = 2.29 − 0.30X_1_ + 0.080X_2_ + 0.040X_3_ + 2.500 × 10^−3^X_1_X_2_ + 0.11X_1_X_3_ + 0.017X_2_X_3_ − 0.32X_1_^2^ − 0.069X_2_^2^ − 0.039X_3_^2^
**7**	Y_7_ = 13.16 − 1.02X_1_ + 0.66X_2_ + 0.084X_3_ − 2.500 × 10^−3^X_1_X_2_ + 0.58X_1_X_3_ + 0.19X_2_X_3_ − 1.79X_1_^2^ − 0.36X_2_^2^ − 0.37X_3_^2^
**8**	Y_8_ = 1.46 + 0.041X_1_ + 0.014X_2_ + 0.030X_3_ + 0.010X_1_X_2_ + 0.078X_1_X_3_ − 2.500 × 10^−3^X_2_X_3_ − 0.098X_1_^2^ − 0.078X_2_^2^ − 0.066X_3_^2^
**9**	Y_9_ = 0.99 − 0.12X_1_ + 0.030X_2_ + 0.024X_3_ − 0.020X_1_X_2_ + 0.038X_1_X_3_ + 5.000 × 10^−3^X_2_X_3_ − 0.13X_1_^2^ − 0.012X_2_^2^ − 4.750 × 10^−3^X_3_^2^
**10**	Y_10_ = 0.59 + 0.000X_1_ + 0.021X_2_ + 6.250 × 10^−3^X_3_ − 0.023X_1_X_2_ + 0.028X_1_X_3_ + 5.000 × 10 ^−3^X_2_X_3_ − 0.088X_1_^2^ − 0.015X_2_^2^ − 5.000 × 10^−3^X_3_^2^
**11**	Y_11_ = 0.13 − 3.750 × 10^−3^X_1_ + 6.250 × 10^−3^X_2_ + 0.000X_3_ − 2.500 × 10^−3^X_1_X_2_ − 5.000 × 10^−3^X_1_X_3_ + 0.000X_2_X_3_ − 9.750 × 10^−3^X_1_^2^ − 4.750 × 10^−3^X_2_^2^ − 2.250 × 10^−3^X_3_^2^

^a^ X_1_, methanol concentration (%); X_2_, liquid/solid ratio (mL/0.5 g); X_3_, extraction time (min); Y_1_–Y_11_ are the measured contents of 11 compounds.

**Table 3:** *F*-test values of regression coefficients, model and lack of fit for Y_1_–Y_11_.

Source		Y_1_	Y_2_	Y_3_	Y_4_	Y_5_	Y_6_	Y_7_	Y_8_	Y_9_	Y_10_	Y_11_
Regression coefficients	X_1_	32.53 **	475.37 **	27.07 **	282.41 **	221.76 **	154.40 **	59.16 **	10.48 *	122.79 **	0.000	7.50 *
	X_2_	4.34	5.32	8.39 *	2.27	5.72 *	11.17 *	24.80 **	1.16	7.52 *	10.22 *	20.83 **
	X_3_	2.460 × 10^−3^	22.37 **	6.449 × 10^−3^	11.62 *	5.96 *	2.79	0.40	5.54	4.71	0.88	0.000
	X_1_X_2_	1.31	0.67	0.43	0.050	0.17	5.452 × 10^−3^	1.786 × 10^−3^	0.31	1.67	5.73 *	1.67
	X_1_X_3_	10.41 *	0.11	12.74 **	3.22	0.23	10.08 *	9.69 *	18.49 **	5.87 *	8.56 *	6.67 *
	X_2_X_3_	1.54	0.027	0.21	0.11	0.12	0.27	1.09	0.019	0.10	0.28	0.000
	X_1_^2^	52.41 **	125.77 **	81.53 **	29.85 **	13.26 **	96.24 **	96.09 **	31.12 **	79.82 **	91.17 **	26.68 **
	X_2_^2^	9.25 *	0.45	8.43 *	0.56	1.19	4.34	3.90	19.72 **	0.66	2.68	6.33 *
	X_3_^2^	0.34	10.11 *	0.16	6.70 *	1.35	1.38	4.12	13.90 **	0.099	0.30	1.42
Model		12.70 **	71.78 **	15.88 **	37.69 **	27.88 **	31.68 **	22.74 **	12.01 **	24.99 **	13.64 **	8.20 **
Lack of fit		1.56	0.036	5.09	1.05	1.10	4.78	6.38	0.20	3.42	1.02	0.42

* Significant (*p* < 0.05); ** Extremely significant (*p* < 0.01); Y_1_–Y_11_ are the measured contents of 11 compounds.

**Table 4:** Predicted values and the results of verification experiment.

-	X_1_ (%)	X_2_ (mL/g)	X_3_ (min)	Contents (mg/g) ^a^											Desirability
				Y_1_	Y_2_	Y_3_	Y_4_	Y_5_	Y_6_	Y_7_	Y_8_	Y_9_	Y_10_	Y_11_	
**Predicted**	76	34.1:1	32.79	5.12	1.08	26.90	0.66	1.20	2.37	13.49	1.43	1.03	0.59	0.14	0.90
**Experimental**	76	34:1	33.00	5.18	1.07	28.20	0.65	1.15	2.43	13.57	1.44	1.01	0.60	0.13	
**Relative Error (%)**				1.17	0.64	4.84	2.22	4.55	2.48	0.60	0.94	2.17	1.39	3.97	

^a^ X_1_, methanol concentration (%); X_2_, liquid/solid ratio (mL/g); X_3_, extraction time (min); Y_1_–Y_11_ are the measured contents of 11 compounds.

**Table 5:** Calibration curves, linear range, LOD and LOQ of 11 reference compounds.

Analytes	Regression Equation	*R*^2^	Linear Range (μg/mL)	LOD (ng/mL)	LOQ (ng/mL)
Echinacoside	y = 12,180.9575x − 3.3677	1.0000	2.41–154	35.5	128.9
Luteolin-7-*O*-rutinoside	y = 13,590.9832x − 2.3474	0.9999	0.95–61	27.0	88.7
Acteoside	y = 15,782.4634x − 20.6464	1.0000	13.30–834	56.2	174.3
Luteolin-7-*O*-glucoside	y = 26,416.3027x − 4.8503	0.9999	0.40–25.5	38.8	107.3
Apigenin-7-*O*-glucuronide	y = 20,583.5424x − 2.8324	0.9998	0.91–58	40.6	115.4
Neobudofficide	y = 15,239.2623x − 1.2074	1.0000	2.52–161.5	24.7	86.5
Linarin	y = 17,508.4610x − 2.2655	1.0000	6.09–390	63.2	218.1
*N*^1^,*N*^5^,*N*^10^-(*E*)-tri-*p*-coumaroylspermidine	y = 18,198.3921x − 18.5268	0.9990	3.91–125	32.6	61.3
Crocin III	y = 73,715.0130x − 19.4425	0.9999	3.97–127	45.8	136.2
Apigenin	y = 40,454x − 0.6379	0.9999	0.98–31.25	35.7	131.8
acacetin	y = 24,216.6307x − 3.9914	0.9996	0.45–14.5	28.4	99.7

## Supplementary Materials

Supplementary Materials are available online.
