# Supplementary material for: Optimization of the Extraction Conditions for Buddleja officinalis Maxim. Using Response Surface Methodology and Exploration of the Optimum Harvest Time
Source: Molecules. 2017 Nov 1;22(11):1877. doi: 10.3390/molecules22111877 (PMC6150163; doi:10.3390/molecules22111877)
Supplement: Supplementary file 1 [file molecules-22-01877-s001.pdf]

Optimization of the Extraction Conditions for *Buddleja officinalis* Maxim Using Response Surface Methodology and Exploration of the Optimum Harvest Time

Guoyong Xie [#], Ran Li [#], Yu Han, Yan Zhu, Gang Wu, and Minjian Qin [*]

Department of Resources Science of Traditional Chinese Medicines, State Key Laboratory of Natural Medicines, China Pharmaceutical University, Nanjing 210009, China.

* Corresponding author. minjianqin@163.com (M.J. Qin).

[#] These authors contributed equally to this work.

Table S1. ANOVA results of eleven responses

	Source	Sum of squares	df	Mean Square	*F*-value	*P*-value Prob>F	Significant
Y_1	Model	5.23	9	0.58	12.70	0.0015	Significant
	X_1	1.49	1	1.49	32.53	0.0007	**
	X_2	0.20	1	0.20	4.34	0.0757	
	X_3	1.125E-004	1	1.125E-004	2.460E-003	0.9618	
	X_1X_2	0.060	1	0.060	1.31	0.2896	
	X_1X_3	0.48	1	0.48	10.41	0.0145	*
	X_2X_3	0.070	1	0.070	1.54	0.2552	
	X_1^2	2.40	1	2.40	52.41	0.0002	**
	X_2^2	0.42	1	0.42	9.25	0.0188	*
	X_3^2	0.015	1	0.015	0.34	0.5798	
	Residual	0.32	7	0.046			
	Lack of fit	0.17	3	0.058	1.56	0.3296	Not significant
	Pure error	0.15	4	0.037			
	Cor Total	5.55	16				
	R^2			0.9423			
	adj. R^2			0.8681			
	CV			4.66			
	Adequate precision			11.348			
Y_2	Model	0.61	9	0.067	71.78	< 0.0001	Significant
	X_1	0.45	1	0.45	475.37	< 0.0001	**
	X_2	5.000E-003	1	5.000E-003	5.32	0.0544	
	X_3	0.021	1	0.021	22.37	0.0021	**
	X_1X_2	6.250E-004	1	6.250E-004	0.67	0.4415	
	X_1X_3	1.000E-004	1	1.000E-004	0.11	0.7537	
	X_2X_3	2.500E-005	1	2.500E-005	0.027	0.8750	
	X_1^2	0.12	1	0.12	125.77	< 0.0001	**
	X_2^2	4.211E-004	1	4.211E-004	0.45	0.5246	
	X_3^2	9.500E-003	1	9.500E-003	10.11	0.0155	*
	Residual	6.575E-003	7	9.393E-004			
	Lack of fit	1.750E-004	3	5.833E-005	0.036	0.9893	Not significant
	Pure error	6.400E-003	4	1.600E-003			

Table S1. *Cont.*

	Cor Total	0.61	16				
	R^2			0.9893			
	adj. R^2			0.9755			
	CV			3.35			
	Adequate precision			24.622			
Y_3	Model	54.31	9	6.03	15.88	0.0007	Significant
	X_1	10.28	1	10.28	27.07	0.0012	**
	X_2	3.19	1	3.19	8.39	0.0231	*
	X_3	2.450E-003	1	2.450E-003	6.449E-003	0.9382	
	X_1X_2	0.16	1	0.16	0.43	0.5321	
	X_1X_3	4.84	1	4.84	12.74	0.0091	**
	X_2X_3	0.078	1	0.078	0.21	0.6634	
	X_1^2	30.97	1	30.97	81.53	< 0.0001	**
	X_2^2	3.20	1	3.20	8.43	0.0229	*
	X_3^2	0.060	1	0.060	0.16	0.7020	
	Residual	2.66	7	0.38			
	Lack of fit	2.11	3	0.70	5.09	0.0750	Not significant
	Pure error	0.55	4	0.14			
	Cor Total	56.97	16				
	R^2			0.9533			
	adj. R^2			0.8933			
	CV			2.42			
	Adequate precision			11.745			
Y_4	Model	0.67	9	0.075	37.69	< 0.0001	Significant
	X_1	0.56	1	0.56	282.41	< 0.0001	**
	X_2	4.513E-003	1	4.513E-003	2.27	0.1758	
	X_3	0.023	1	0.023	11.62	0.0113	*
	X_1X_2	1.000E-004	1	1.000E-004	0.050	0.8290	
	X_1X_3	6.400E-003	1	6.400E-003	3.22	0.1160	
	X_2X_3	2.250E-004	1	2.250E-004	0.11	0.7465	
	X_1^2	0.059	1	0.059	29.85	0.0009	**
	X_2^2	1.112E-003	1	1.112E-003	0.56	0.4791	
	X_3^2	0.013	1	0.013	6.70	0.0361	*
	Residual	0.014	7	1.989E-003			
	Lack of fit	6.125E-003	3	2.042E-003	1.05	0.4632	Not significant
	Pure error	7.800E-003	4	1.950E-003			
	Cor Total	0.69	16				
	R^2			0.9798			
	adj. R^2			0.9538			
	CV			8.92			
	Adequate precision			18.636			
Y_5	Model	1.32	9	0.15	27.88	0.0001	Significant
	X_1	1.16	1	1.16	221.76	< 0.0001	**
	X_2	0.030	1	0.030	5.72	0.0480	*
	X_3	0.031	1	0.031	5.96	0.0447	*

Table S1. *Cont.*

	X_1X_2	9.000E-004	1	9.000E-004	0.17	0.6911	
	X_1X_3	1.225E-003	1	1.225E-003	0.23	0.6436	
	X_2X_3	6.250E-004	1	6.250E-004	0.12	0.7401	
	X_1^2	0.070	1	0.070	13.26	0.0083	**
	X_2^2	6.241E-003	1	6.241E-003	1.19	0.3114	
	X_3^2	7.078E-003	1	7.078E-003	1.35	0.2834	
	Residual	0.037	7	5.244E-003			
	Lack of fit	0.017	3	5.542E-003	1.10	0.4450	Not significant
	Pure error	0.020	4	5.020E-003			
	Cor Total	1.35	16				
	R^2			0.9729			
	adj. R^2			0.9380			
	CV			7.28			
	Adequate precision			16.048			
Y_6	Model	1.31	9	0.15	31.68	< 0.0001	Significant
	X_1	0.71	1	0.71	154.40	< 0.0001	**
	X_2	0.051	1	0.051	11.17	0.0124	*
	X_3	0.013	1	0.013	2.79	0.1387	
	X_1X_2	2.500E-005	1	2.500E-005	5.452E-003	0.9432	
	X_1X_3	0.046	1	0.046	10.08	0.0156	*
	X_2X_3	1.225E-003	1	1.225E-003	0.27	0.6212	
	X_1^2	0.44	1	0.44	96.24	< 0.0001	**
	X_2^2	0.020	1	0.020	4.34	0.0757	
	X_3^2	6.322E-003	1	6.322E-003	1.38	0.2787	
	Residual	0.032	7	4.586E-003			
	Lack of fit	0.025	3	8.367E-003	4.78	0.0824	Not significant
	Pure error	7.000E-003	4	1.750E-003			
	Cor Total	1.34	16				
	R^2			0.9760			
	adj. R^2			0.9452			
	CV			3.24			
	Adequate precision			16.125			
Y_7	Model	28.66	9	3.18	22.74	0.0002	Significant
	X_1	8.28	1	8.28	59.16	0.0001	**
	X_2	3.47	1	3.47	24.80	0.0016	**
	X_3	0.056	1	0.056	0.40	0.5468	
	X_1X_2	2.500E-005	1	2.500E-005	1.786E-004	0.9897	
	X_1X_3	1.36	1	1.36	9.69	0.0170	*
	X_2X_3	0.15	1	0.15	1.09	0.3319	
	X_1^2	13.45	1	13.45	96.09	< 0.0001	**
	X_2^2	0.55	1	0.55	3.90	0.0889	
	X_3^2	0.58	1	0.58	4.12	0.0820	
	Residual	0.98	7	0.14			
	Lack of fit	0.81	3	0.27	6.38	0.0527	Not significant
	Pure error	0.17	4	0.042			

Table S1. *Cont.*

	Cor Total	29.64	16				
	R^2			0.9669			
	adj. R^2			0.9244			
	CV			3.12			
	Adequate precision			14.108			
Y_8	Model	0.14	9	0.016	12.01	0.0017	Significant
	X_1	0.014	1	0.014	10.48	0.0143	*
	X_2	1.513E-003	1	1.513E-003	1.16	0.3164	
	X_3	7.200E-003	1	7.200E-003	5.54	0.0508	
	X_1X_2	4.000E-004	1	4.000E-004	0.31	0.5963	
	X_1X_3	0.024	1	0.024	18.49	0.0036	**
	X_2X_3	2.500E-005	1	2.500E-005	0.019	0.8936	
	X_1^2	0.040	1	0.040	31.12	0.0008	**
	X_2^2	0.026	1	0.026	19.72	0.0030	**
	X_3^2	0.018	1	0.018	13.90	0.0074	**
	Residual	9.095E-003	7	1.299E-003			
	Lack of fit	1.175E-003	3	3.917E-004	0.20	0.8929	Not significant
	Pure error	7.920E-003	4	1.980E-003			
	Cor Total	0.15	16				
	R^2			0.9392			
	adj. R^2			0.8610			
	CV			2.69			
	Adequate precision			9.124			
Y_9	Model	0.22	9	0.024	24.99	0.0002	Significant
	X_1	0.12	1	0.12	122.79	< 0.0001	**
	X_2	7.200E-003	1	7.200E-003	7.52	0.0288	*
	X_3	4.513E-003	1	4.513E-003	4.71	0.0666	
	X_1X_2	1.600E-003	1	1.600E-003	1.67	0.2372	
	X_1X_3	5.625E-003	1	5.625E-003	5.87	0.0459	*
	X_2X_3	1.000E-004	1	1.000E-004	0.10	0.7561	
	X_1^2	0.076	1	0.076	79.82	< 0.0001	**
	X_2^2	6.318E-004	1	6.318E-004	0.66	0.4434	
	X_3^2	9.500E-005	1	9.500E-005	0.099	0.7620	
	Residual	6.705E-003	7	9.579E-004			
	Lack of fit	4.825E-003	3	1.608E-003	3.42	0.1328	Not significant
	Pure error	1.880E-003	4	4.700E-004			
	Cor Total	0.22	16				
	R^2			0.9698			
	adj. R^2			0.9310			
	CV			3.36			
	Adequate precision			15.324			
Y_{10}	Model	0.043	9	4.824E-003	13.64	0.0012	Significant
	X_1	0.000	1	0.000	0.000	1.0000	
	X_2	3.613E-003	1	3.613E-003	10.22	0.0151	*
	X_3	3.125E-004	1	3.125E-004	0.88	0.3785	
	X_1X_2	2.025E-003	1	2.025E-003	5.73	0.0479	*

Table S1. Cont.

	X_1X_3	3.025E-003	1	3.025E-003	8.56	0.0222	*
	X_2X_3	1.000E-004	1	1.000E-004	0.28	0.6113	
	X_1^2	0.032	1	0.032	91.17	< 0.0001	**
	X_2^2	9.474E-004	1	9.474E-004	2.68	0.1457	
	X_3^2	1.053E-004	1	1.053E-004	0.30	0.6023	
	Residual	2.475E-003	7	3.536E-004			
	Lack of fit	1.075E-003	3	3.583E-004	1.02	0.4709	Not significant
	Pure error	1.400E-003	4	3.500E-004			
	Cor Total	0.046	16				
	R^2			0.9461			
	adj. R^2			0.8767			
	CV			3.49			
	Adequate precision			11.008			
Y_{11}	Model	1.107E-003	9	1.230E-004	8.20	0.0056	Significant
	X_1	1.125E-004	1	1.125E-004	7.50	0.0290	*
	X_2	3.125E-004	1	3.125E-004	20.83	0.0026	**
	X_3	0.000	1	0.000	0.000	1.0000	
	X_1X_2	2.500E-005	1	2.500E-005	1.67	0.2377	
	X_1X_3	1.000E-004	1	1.000E-004	6.67	0.0364	*
	X_2X_3	0.000	1	0.000	0.000	1.0000	
	X_1^2	4.003E-004	1	4.003E-004	26.68	0.0013	**
	X_2^2	9.500E-005	1	9.500E-005	6.33	0.0400	*
	X_3^2	2.132E-005	1	2.132E-005	1.42	0.2721	
	Residual	1.050E-004	7	1.500E-005			
	Lack of fit	2.500E-005	3	8.333E-006	0.42	0.7510	Not significant
	Pure error	8.000E-005	4	2.000E-005			
	Cor Total	1.212E-003	16				
	R^2			0.9133			
	adj. R^2			0.8019			
	CV			3.12			
	Adequate precision			7.406			

* Significant (P<0.05);

** Extremely significant (P<0.01).

Y_1~Y_{11} are the measured contents of 11 compounds.

Table S2. The content of 11 bioactive compounds in *B. officinalis* at different growth stages (Mean±SD, mg/g, n=3)

Analyte	S1	S2	S3	S4
**Echinacoside**	8.03±0.10	5.79±0.04	9.37±0.02	5.53±0.17
**Luteolin-7-O-rutinoside**	0.29±0.00	0.42±0.02	0.38±0.01	0.20±0.00
**Acteoside**	69.11±0.71	52.06±0.78	69.34±0.33	27.99±0.78
**Luteolin-7-O-glucoside**	0.69±0.01	0.69±0.03	0.57±0.01	0.44±0.01

Table S2. *Cont.*

Apigenin-7-O-glucuronide	**1.74±0.01**	**1.38±0.01**	**1.92±0.07**	**1.15±0.01**
**Neobudofficide**	1.28±0.01	1.04±0.03	1.47±0.02	0.56±0.00
Linarin	20.02±0.24	19.92±0.90	22.43±0.49	11.59±0.15
***N^1, N^5, N^{10}*-(E)-tri-*p*-Coumaroylspermidine**	2.80±0.02	2.31±0.11	1.55±0.01	0.91±0.02
**Crocin Ⅲ**	0.24±0.00	1.51±0.04	2.37±0.10	1.01±0.01
**Apigenin**	0.47±0.01	0.23±0.00	0.36±0.02	1.26±0.01
**Acacetin**	0.05±0.00	0.06±0.00	0.14±0.00	0.73±0.01
